# Production of codon-optimized Factor C fragment from *Tachypleus gigas* in the *Pichia pastoris* GS115 expression system for endotoxin detection

**DOI:** 10.1186/s43141-023-00557-y

**Published:** 2023-10-17

**Authors:** Zubaidi Bachtiar, Apon Zaenal Mustopa, Rika Indri Astuti, Fauziyah Fauziyah, Fatimah Fatimah, Rozirwan Rozirwan, Tuah Nanda Merlia Wulandari, Dina Permata Wijaya, Fitri Agustriani, Arwansyah Arwansyah, Herman Irawan, Jendri Mamangkey

**Affiliations:** 1 Post Graduate Program of Biotechnology, Bogor Agricultural University, Bogor, Indonesia; 2 Research Center for Genetic Engineering, Research Organization for Life Sciences and Environment, National Research and Innovation Agency (BRIN), KST Soekarno, Cibinong, Bogor 16911 Indonesia; 3 Department of Biology, IPB University, Bogor, West Java 16680 Indonesia; 4 Biotechnology Research Center, IPB University, Bogor, West Java Indonesia; 5 Marine Science Study Program, Faculty of Mathematics and Natural Science, University of Sriwijaya, Palembang, Indonesia; 6 Research Center for Conservation of Marine and Inland Water Resources, National Research and Innovation Agency, Cibinong, 16911 Indonesia; 7 Department of Pharmacy, Faculty of Science, Sriwijaya University, Indralaya, South Sumatera Indonesia; 8 Department of Chemistry Education, Faculty of Teacher Training and Education, Tadulako University, Palu, Indonesia; 9 Department of Biology Education, Faculty of Education and Teacher Training, Universitas Kristen Indonesia, Jl. Mayjen Sutoyo No. 2, Cawang, Jakarta Timur 13630 Jakarta, Indonesia

**Keywords:** Tachypleus gigas, Endotoxin, Factor C, pPIC9K

## Abstract

**Background:**

Factor C (FC) is widely used as a standard material for endotoxin testing. It functions as a zymogenic serine protease and serve as a biosensor that detects lipopolysaccharides. Prior investigations involving molecular docking and molecular dynamics simulations of FC demonstrated an interaction between the C-type lectin domain (CLECT) and the ligand lipopolysaccharide (lipid A). In this study, our aim was to assess the stability of the interaction between fragment FC and the lipid A ligand using protein modeling approaches, molecular docking, molecular dynamics simulation, and gene construction into the pPIC9K expression vector.

**Methods and results:**

The FC structure was modelled by online tools. In this case, both molecular docking and MD simulations were applied to identify the interaction between protein and ligand (lipid A) including its complex stability. The FC structure model using three modeling websites has varied values, according to a Ramachandran plot study. When compared to other models, AlphaFold server modeling produced the best Ramachandran findings, with residues in the most advantageous area at 88.3%, followed by ERRAT values at 89.83% and 3D Verify at 71.93%. From the docking simulation of FC fragments with three ligands including diphosphoryl lipid A, FC-Core lipid A, and Kdo2 lipid A can be an activator of FC protein by binding to receptor regions to form ligand-receptor complexes. MD simulations were performed on all three complexes to assess their stability in water solvents showing that all complexes were stable during the simulation. The optimization of recombinant protein expression in *Pichia pastoris* was conducted by assessing the OD value and protease activity. Induction was carried out using 1% (v/v) methanol in BMMY media at 30°C for 72 h.

**Conclusions:**

Protein fragments of Factor C has been proven to detect endotoxins and serve as a potential biomarker. Molecular docking simulation and MD simulation were employed to study the complex formation of protein fragments FC with ligands. The expression of FC fragments was successfully achieved through heterologous expression. We propose optimizing the expression of FC fragments by inducing them with 1% methanol at 30°C and incubating them for 72 h. These optimized conditions are well-suited for upscaling the production of recombinant FC fragments using a bioreactor.

## Background

Horseshoe crabs are exotic aquatic organisms belonging to the family Limulidae and are considered living fossil animals [1]. Currently, there are four known species of horseshoe crabs in the world, namely *Carcinoscorpius rotundicauda* [2], *Tachypleus gigas* [3], *Tachypleus tridentatus* [4], and *Limulus polyphemus* [5]. Among these, *Carcinoscorpius rotundicauda, Tachypleus gigas*, and *Tachypleus tridentatus* are commonly found in coastal areas of Indonesia, including Indonesia. They are collectively referred to as Asian horseshoe crabs. On the other hand, *Limulus polyphemus*, also known as the Atlantic horseshoe crab, is exclusively found along the Atlantic coast of North America [6].

Asian horseshoe crabs, specifically *C. rotundicauda* and *T. gigas* are protected animals in Indonesia, although their conservation status remains unclear and insufficient. These species have been categorized as Near Threatened (2010), Threatened (2014), and Data Deficient [7]. The “Data Deficient” category indicates the unavailability of adequate data to determine estimates of extinction risk, distribution, and population numbers. According to the Minister of Environment’s Regulation Number P.92/MENLHK/SETJEN/KUM.1/8/2018, all three types of horseshoe crabs are classified as protected animals [8]. Even though their conservation status seems contradictory, the Atlantic horseshoe crab or *L. polyphemus* produces *Limulus Amoebocyte Lysate* (LAL) in their bloodstream which is known as the current Factor C used as a standard material for endotoxin testing. Proenzyme serine proteases are the first proteins in the cascade reaction that lead to LAL coagulation upon contact with the endotoxin [9]. *Tachypleus* sp. produces *Tachyplesin amoebocyte lysate* (TAL) and *C. rotundicauda* produces *Carcinoscorpius amoebocyte lysate* (CAL) which can also be used to detect gram bacterial endotoxins negative, human blood endotoxins, and pathogens in medicinal products [10].

Gram-negative bacteria’s outer membrane is made of lipopolysaccharide (LPS), a complex macromolecule with three structural parts: core oligosaccharides, lipid A, and O-antigens [11]. LPS within the outer membrane has the ability to activate Factor C through autocatalysis, a protein responsible for endotoxins binding. Upon binding to endotoxins, Factor C interacts with serine protease to remain active [12, 13]. Once activated by LPS, Factor C activates coagulation Factor B, which subsequently converts pro-clotting enzymes into active clotting enzymes. These enzymes then facilitate the conversion of proteolytic coagulants to coagulants, resulting in the spontaneous formation of insoluble polymers. In this manner, Factor C functions by binding to defense molecules from pathogens (PAMPs) such as LPS present on the cell walls of Gram-negative bacteria. This process ultimately forms a barrier at the site of microbial invasion [14].

Factor C is synthesized as a single polypeptide has a molecular weight of 123 kDa. It consists of two polypeptide chains connected by disulfide bonds: a heavy chain (80 kDa) and a light chain, (43 kDa) in *T. tridentatus*, while in *C. rotundicauda*, it consists of a heavy chain (80 kDa) and a light chain (52 kDa) [13, 15–17]. Factor C of *C. rotundicauda* has been cloned and expressed in *Escherichia coli* [18]. Muta et al. [12] cloned the Factor C-coding gene of the Japanese horseshoe crab (*T. tridentatus*) in two separates, overlapping partial fragments. Recombinant Factor C (rFC) has been expressed in different host cells, including, among others: *Pichia pastoris* [19, 20], *Saccharomyces cerevisiae* [21], mammalian cells [22, 23], and insect cells using the baculovirus system [24]. Factor C has also been cloned from the species *L. polyphemus* [25]. Our report on the analysis of docking simulations in silico and molecular dynamics of protein Factor C (Receptor) simulations with ligands of lipid A (lipopolysaccharides) shows that the active sites and binding pockets between proteins and lipopolysaccharide ligands of gram-negative bacteria, are present in Heavy Chain Fragment Factor C with Lectin Domain [26]. In this study, cloning and expression of Factor C gene fragments from *Tachypleus gigas* in the eukaryotic organism *Pichia pastoris* was carried out.

## Methods

The Factor C gene from Horseshoe crab (*T. gigas*) has been analyzed for its active site and binding site on certain amino acid parts cut for recombinant protein studies by ligating Factor C gene fragments into the pPIC9K plasmid which is a plasmid derived from yeast. *E. coli* DH5α bacteria are used as hosts for cell cloning. The cloning of recombinant plasmids is then cut on the side of the SacI restriction enzyme section to neutralize the recombinant plasmids so that they can enter and integrate into the expression host. Yeast with strain GS115 hosts expression of Factor C gene fragments.

Gene Factor C fragment expression was analyzed using SDS-PAGE. Bioinformatic studies were conducted by modeling protein from Factor C gene fragments using a web server, followed by protein model validation with Ramachandran, 3D verify, ERRAT, molecular docking to analyze the interaction between proteins and ligands, and MD simulation to determine the stability of molecular docking results with a simulation time of 100 ns.

### Physicochemical analysis of factor C protein fragment

The gene sequence was translated using SnapGene to obtain amino acid data. The amino acid sequence was then analyzed for its physical and chemical parameters using the ExPASy—ProtParam tool, a web server. This program predicted the hydrophobicity of the amino acid sequence, isoelectric point, and molecular weight of the protein. Additionally, the protein solubility was analyzed using the Protein-sol sequence solubility web server (Protein-sol sequence solubility (manchester.ac.uk) [27].

### Protein modeling analysis and molecular docking

Protein modeling was initiated by entering the files (fasta) into several web-based servers including Alphafold (AlphaFold2.ipynb-Colaboratory (google.com), Robetta (https://robetta.bakerlab.org), and I-Tasser (https://zhanggroup.org/I-TASSER/). A web-based protein refine model server or Galaxy refine (GalaxyWEB (seoklab.org) was used to correct some missing residues. The results of the refined protein model were validated using several validity parameters including Ramachandran Plot, 3D Verify Structure, and ERRAT (www.saves.mbi.ucla.edu). Investigation on the protein fragment Factor C as a receptor to obtain ligand lipid A binding pockets, we simulated molecular docking using a web-based hdock server. Ligands were retrieved from the Pubchem database. The three ligand component structures were then downloaded in SDF form. The SDF file is then converted into a PDB file using OpenBabel 2.4.1 Program Packages. Geometry optimization on ligands was carried out using Avogadro software. Docking simulations were performed with Factor C fragment proteins as receptors to obtain binding pockets and lipid A as ligands.

The website, http://hdock.phys.hust.edu.cn/ was used to execute HDOCK-based molecular docking [28]. The docking parameters were calculated using the docking online tool’s defaults. Then, Pymol software and PLIP online tools were used to visualize the interaction between the protein and its ligands. The bond stability between receptor proteins and lipid A ligands resulting from molecular docking, molecular dynamics simulations were carried out for 100 ns using AMBER 20 program, the best modeling outcomes will be confirmed. The methodologies for molecular docking and MD modeling are identical to those used in our earlier work, which was described in the previous study [29].

### Molecular dynamics simulation

To assess the stability of the protein-ligand complex within a TIP3P water solvent, an all-atom molecular dynamics (MD) simulation was conducted using the Amber20 software package [30]. The protein’s force field parameters were determined using the AMBER14 force field, while the ligand’s parameters were computed using the general amber force field (GAFF) [31]. Electrostatic interactions and hydrogen bonds were managed using the Particle Mesh Ewald (PME) algorithm [32] and the SHAKE algorithm [33]. The cut-off distance and time step were set to 10 Å and 2 fs, respectively, for the entire system. The MD simulation involved multiple steps: energy minimization, followed by heating the system from 0 to 300 K over 2 ns using the NVT ensemble. To maintain system temperature and pressure, the Langevin thermostat [34] and the isotropic position scaling algorithm were applied during the simulation. Subsequently, the NPT ensemble was employed to equilibrate the system for 100 ns. Trajectories were collected at intervals of 5000 steps for each MD simulation. The CPPTRAJ software package was used to analyze the MD trajectories [35]. The MD simulation protocols closely followed our previous studies [36, 37].

### Cloning preparation of gene fragment Factor C

Factor C gene sequences have been carried out active site topology analysis and pocket binding in previous studies, obtained sequences of Factor C (Lectin-serine) gene fragments that play an important role in silico Factor C studies for protein expression analysis. The sequence of these fragments was assembled in silico and we make the gene into a synthetic gene that has been inserted into the plasmid. pPIC9K was selected to be the expression vector in the host *Pichia pastoris* cell strain GS115. The gene cloning stage is introduced into *E. coli* DH5α (competent cells). *E. coli* transformants were selected on LB agar media supplemented with 20 μL of kanamycin per plate.

### Linearization of recombinant plasmids

Positive clones (plasmids carrying genes) were grown on 5 mL of liquid LB media for isolation using plasmid Midi Kit (Qiagen) protocol. The results of plasmid isolation were linearized using the restriction enzyme SacI. The linearization results were verified using 1% agarose gel (marker 1 kb). Through the use of QIAquick PCR Purification Kit (Qiagen) procedure, the generated DNA bands were purified. Plasmids that had been purified were kept at −20°C for further study.

### Transformation of recombinant plasmids with Pichia pastoris GS115

The linearized recombinant plasmids were then introduced into *P. pastoris* GS115 cells (competent cells) by electroporation method. A total of 80 μl of competent *P. pastoris* GS115 cells were mixed with 5 μg of linear plasmids in a microtube. Competent cells and plasmids are inserted into electroporated cuvettes (2 mm gap) that have been cooled at −4°C. The sample was incubated in ice for 5 min. Electroporation was carried out under conditions of 2 kV400 Ω on the electroporator machine. After electroporation, 1 ml of cold sorbitol solution (1 M) was added to the cuvette, the contents of the cuvette were transferred into a 1.5-mL sterile microtube and shaker incubator at 30°C for 2 h. A total of 100, 150, and 200 μL of transformed *P. pastoris* GS115 cells were spread on YPDS agar media containing 0.25 mg/ml of geneticin on the plate incubated for 2–3 days at 30°C until colonies formed. The colonies formed were purified by scratching method on fresh YPD or YPDS agar media containing geneticin.

### Expression of Factor C gene fragment in Pichia pastoris

Single colonies of recombinant *P. pastoris* GS115 were cultured overnight in BMGY medium at 30°C under agitation of 250×*g* until reaching the logarithmic phase (OD_600_ = 2–6). The culture was then centrifuged for 5 min at 1500×*g* and 4°C. The obtained pellet was resuspended in 25 mL of BMMY medium supplemented with 0.5% methanol as an inducer to achieve an OD_600_ of 1.0. Induction was repeated during a 24-h cultivation period. Harvesting was performed by centrifugation for 5 min at room temperature and 1500×*g* to obtain the supernatant containing Factor C protein fragment. Subsequently, SDS-PAGE was carried out to analyze the successful expression of Factor C in *P. pastoris* GS115.

### Optimization of culture conditions

Selected colonies from the prior results were then expressed in a shake-flask fermentation with aeration (1:10). The recombinant yeasts were grown in buffered glycerol complex medium (BMGY) to achieve high biomass production. The culture was then transferred to buffered methanol complex medium (BMMY) to determine the optimal methanol concentration. Methanol was added to the expression medium at final concentrations of 0.5%, 1%, and 2% (v/v) and incubated at various temperatures (25°C, 30°C, 37°C) with a shaker speed of 250×*g* for 24 h. The kinetics of growth were analyzed by measuring the OD_600_ value every 24 h until 96 h.

### SDS-PAGE analysis

Acrylamide gel electrophoresis was performed using a separating gel (12%) composed of 3.2 mL of ddH2O, 4 mL of 30% acrylamide, 2.6 mL of 1.5 M Tris-HCl pH 8.8, 100 µL of 10% sodium dodecyl sulfate (SDS), 100 µL of 10% ammonium persulfate (APS), and 10 µL of TEMED. All reagents were thoroughly mixed, and 5 mL of the mixture was poured into the gel mold. Then, 1 mL of 70% ethanol was added. The separating gel was allowed to polymerize for 20–30 min until solidified. After solidification, the ethanol layer on top of the separating gel was discarded. For the stacking gel (4%), 2.93 mL of ddH2O, 0.67 mL of 30% acrylamide, 1.3 mL of 0.5 M Tris-HCl pH 6.8, 50 µL of 10% SDS, 500 µL of 10% APS, and 5 µL of TEMED were mixed to ensure homogeneous mixing. A total of 30 µL of the sample was mixed with 15 µL of loading buffer in a 1.5-mL tube. The sample was then denatured at 95°C for 10 min. Subsequently, 30 µL of the sample and 5 µL of the marker were loaded into the gel wells. Electrophoresis was run at voltages of 80 V and 110 V for 130 min. The SDS-PAGE gel was stained with Coomassie brilliant blue (CBB) and incubated overnight. The gel was then rinsed (destained) until clear protein bands appeared.

### Zymogram

According to Bencsik et al. [38], the zymogram test contained 8% (v/v) separating gel and 4.5% (v/v) stacking gel with a 1.5% casein addition. The gel is incubated in 2.5% Triton X-100 at room temperature for 1 h following the separation procedure. The Gel was then treated with a 10 mM pH 8 Tris HCl buffer for an overnight period. For 2 to 3 h, the gel was dyed with Sigma’s 0.05% Coomassie brilliant blue G-250. The next step was rinse (destained) the sample until clear protein bands were visible.

### Protease activity test

Protease activity was measured using a modified version of Cupp-Enyard’s [39] technique. In a 96-well microplate, the protease test was performed at 540 nm.

## Results

### Solubility of Factor C protein

The solubility of the recombinant protein was analyzed using the Protparam Expasy webserver, which yielded an isoelectric point (pI) of 6.57. Proteins with isoelectric point (pI) values falling between 5 and 7 are classified as exhibiting pronounced solubility. Moreover, a greater abundance of negatively charged amino acid residues corresponds to an elevated degree of protein solubility [37]. The recombinant protein’s isoelectric point falls within the 5–7 range, confirming its elevated solubility characteristics.

### Factor C gene fragment modeling

**Fig. 1:**
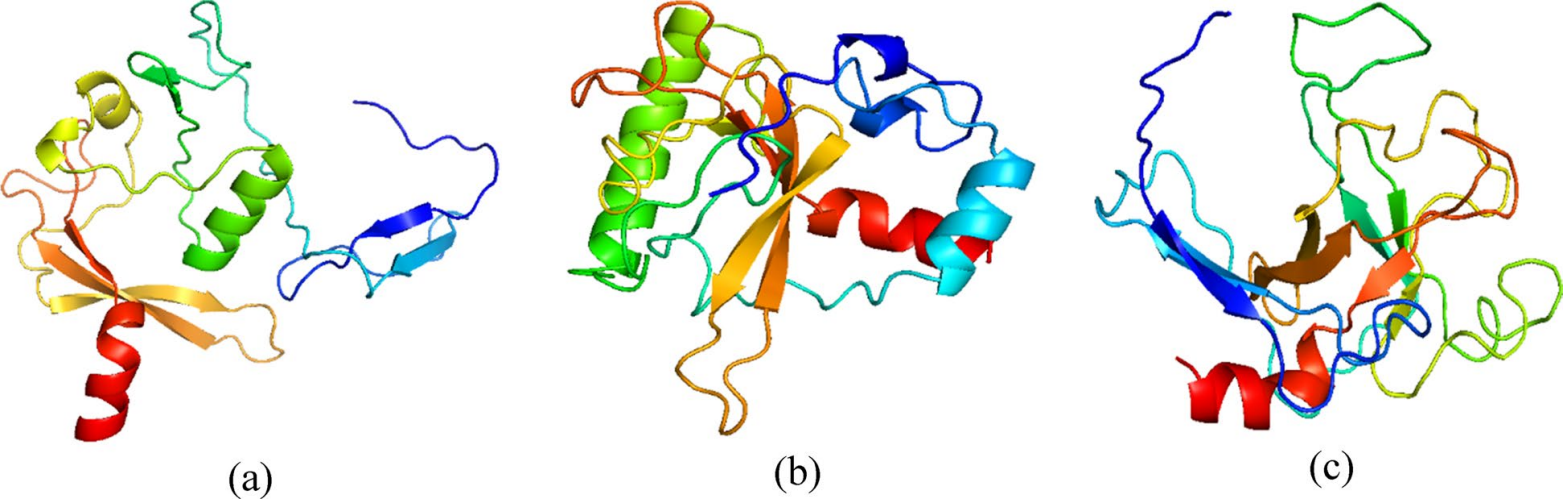
3D Structure of lectin-serine protease Factor C modeled using **a** alphafold, **b** robetta, and **c** i-tasser

The results of the three-dimensional protein structure modeling display the protein structure of the Factor C fragment using three different web servers: Alphafold, Robetta, and I-Tasser. The models presented by these three protein modeling servers consist of α-helices, β-sheets, and loop/coil structures. The three-dimensional protein model structures can be observed in Fig. 1.

**Fig. 2:**
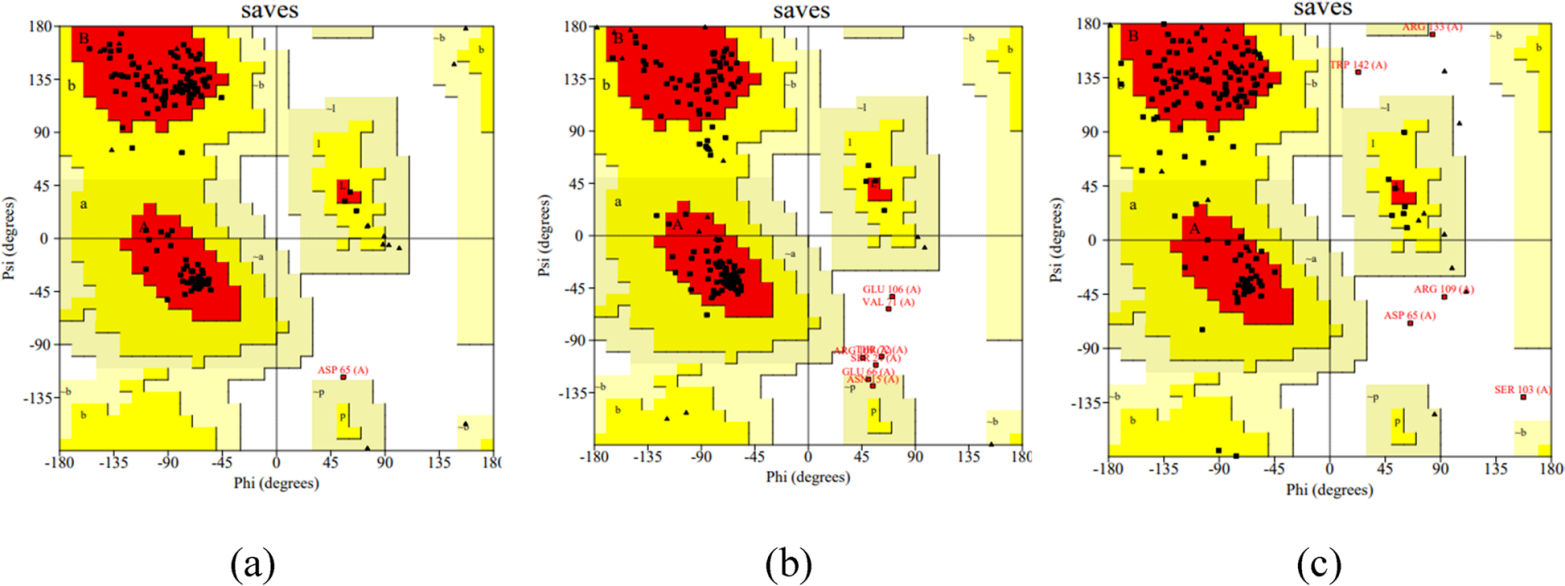
Ramachandran plot analysis of *T. gigas* Factor C **a** model by alphafold, **b** model by robetta, and **c** model by i-tasser

One of the most fundamental ideas in structural biology is the Ramachandran plot, which is frequently applied to validate protein structure models. On Fig. 2, a Ramachandran plot analysis of the *T. gigas* Factor C protein structure is presented in this work

AlphaFold modeling exhibits the best Ramachandran results when compared to other models because it has residues in the most advantageous region of 95.8% (quadrant 1), the allowable additional region of 3.5% (quadrant 2), the generously permitted region of 0.0% (quadrant 3), and the area that is not permitted of 0.7% (quadrant 4). Ramachandran plot analysis demonstrates that Factor C structure model with three modeling websites (Fig. 2) has different values. Better quality and stability of protein structure are correlated with higher percentages of amino acid residues in the most favored area and lower percentages of residue in the not authorized region [37]. The AlphaFold modeling of the *T. gigas* Factor C structure reveals that it has a medium quality and a tendency to be stable. In this case, when a protein structure has a preferred region score of >90% and a forbidden area score of 2%, it is said to be of excellent quality [40].

Validation analysis of the 3D model structure was also performed using the ERRAT and Verify 3D scores for the Factor C protein fragment. ERRAT serves as an overall quality factor for non-bonded atom interactions, indicating that higher scores correspond to better protein quality. High-resolution structures typically have ERRAT scores above 95% and Verify 3D scores exceeding 80% (Table 2).

### Molecular docking

**Fig. 3:**
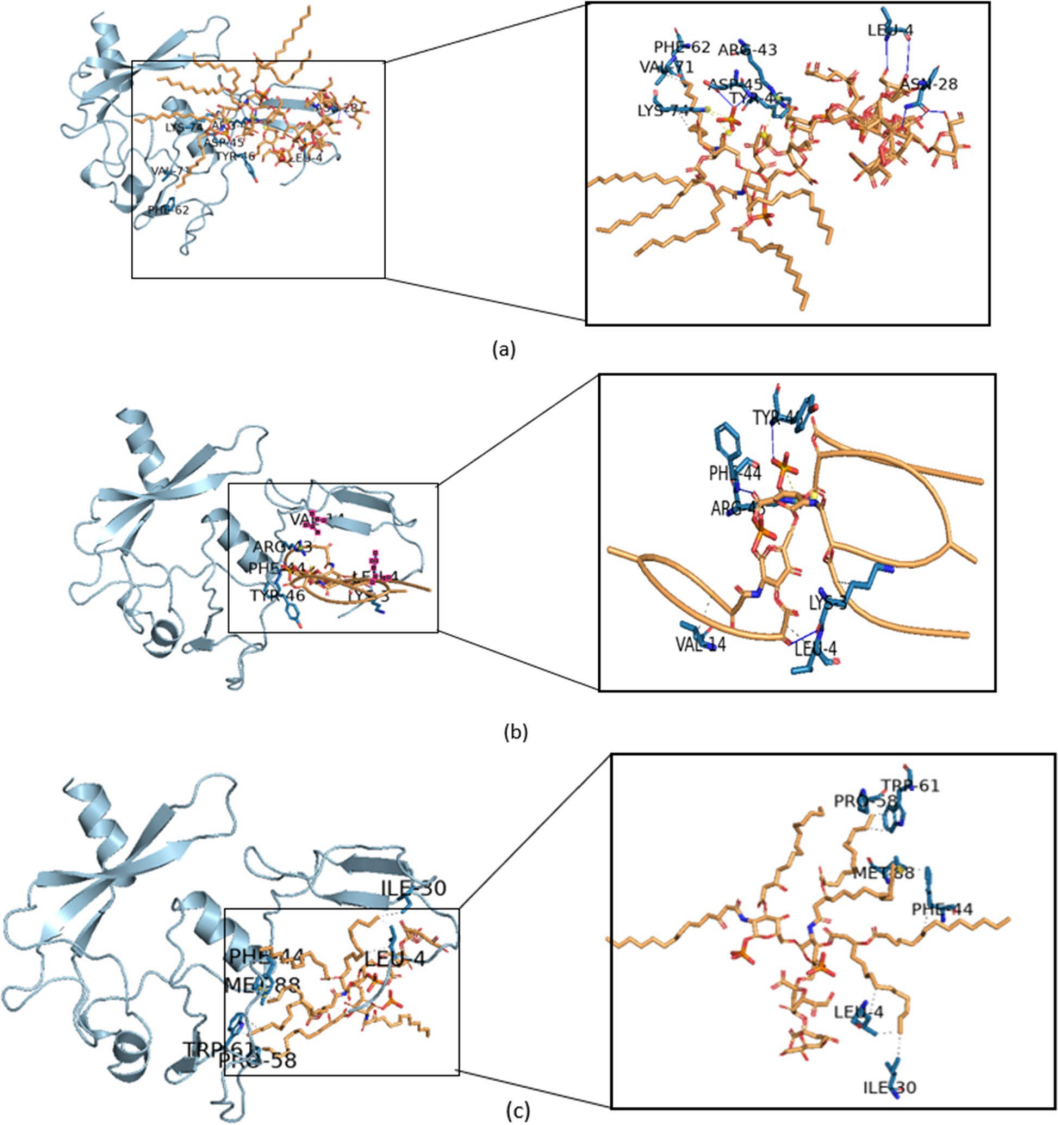
Binding of the ligand to protein Factor C receptor site. **a** Complex 1 (protein-core lipid A ligand), **b** Complex 2 (protein-diphosphoryl lipid A ligand), and **c** complex 3 (protein-Kdo2 lipid A ligand). The conformational poses of each complex were visualized with the pymol program

Molecular docking simulation is a method used to identify the binding site of active compounds to the protein's catalytic site [41]. This method is advantageous and widely applied in the design of new drugs as it can elucidate how specific small molecules bind to proteins using computational algorithms and scoring functions [42]. The docking results explain how the ligand can bind to the receptor protein with several observed interactions of amino acid residues, as shown in Fig. 3.

Some amino acid residues that bind to the receptor protein with the ligand lipid A in complex 1 (core lipid A) include Phe22, Ile30, Ser25, Tyr46, Ser48, Tyr70, Ser73, Lys74, Asp127, Arg129, and Arg132. In complex 2 (diphosphoryl lipid A), the amino acid residues Met1 and Tyr46 are involved in binding, and in complex 3 (Kdo2 lipid A), the binding involves the amino acid residues Gly2, Phe44, Tyr46, Ser49, and Asp59. All three complexes exhibit the same binding pattern with the amino acid residue Tyr46. The interaction results of the amino acid residues from the three complexes indicate that the receptor protein with the lipid A ligand forms hydrogen interactions Tables 1 and 2.

**Table 1:** Solubility of Factor C protein fragment with isoelectric point

**Amino acid**	**Composition**	**Percentage**
Ala (A)	11	6.40%
Arg (R)	10	5.80%
Asn (N)	8	4.70%
Asp (D)	11	6.40%
Cys (C)	5	2.90%
Gln (Q)	4	2.30%
Glu (E)	8	4.70%
Gly (G)	19	11.10%
His (H)	1	0.60%
Ile (I)	6	3.50%
Leu (L)	13	7.60%
Lys (K)	9	5.30%
Met (M)	2	1.20%
Phe (F)	8	4.70%
Pro (P)	6	3.50%
Ser (S)	21	12.30%
Thr (T)	6	3.50%
Trp (W)	5	2.90%
Tyr (Y)	4	2.30%
Val (V)	14	8.20%
Pyl (O)	0	0.00%
Sec (U)	0	0.00%

**Table 2:** Quality evaluation of Factor C protein structure with ERRAT and verify 3D webserver

**Modelling structure of Factor C**	**ERRAT (%)**	**Verify 3D (%)**
AlphaFold	89.83	71.93
Robetta	89.79	73.10
I-Tasser	78.76	60.23

**Fig. 4:**
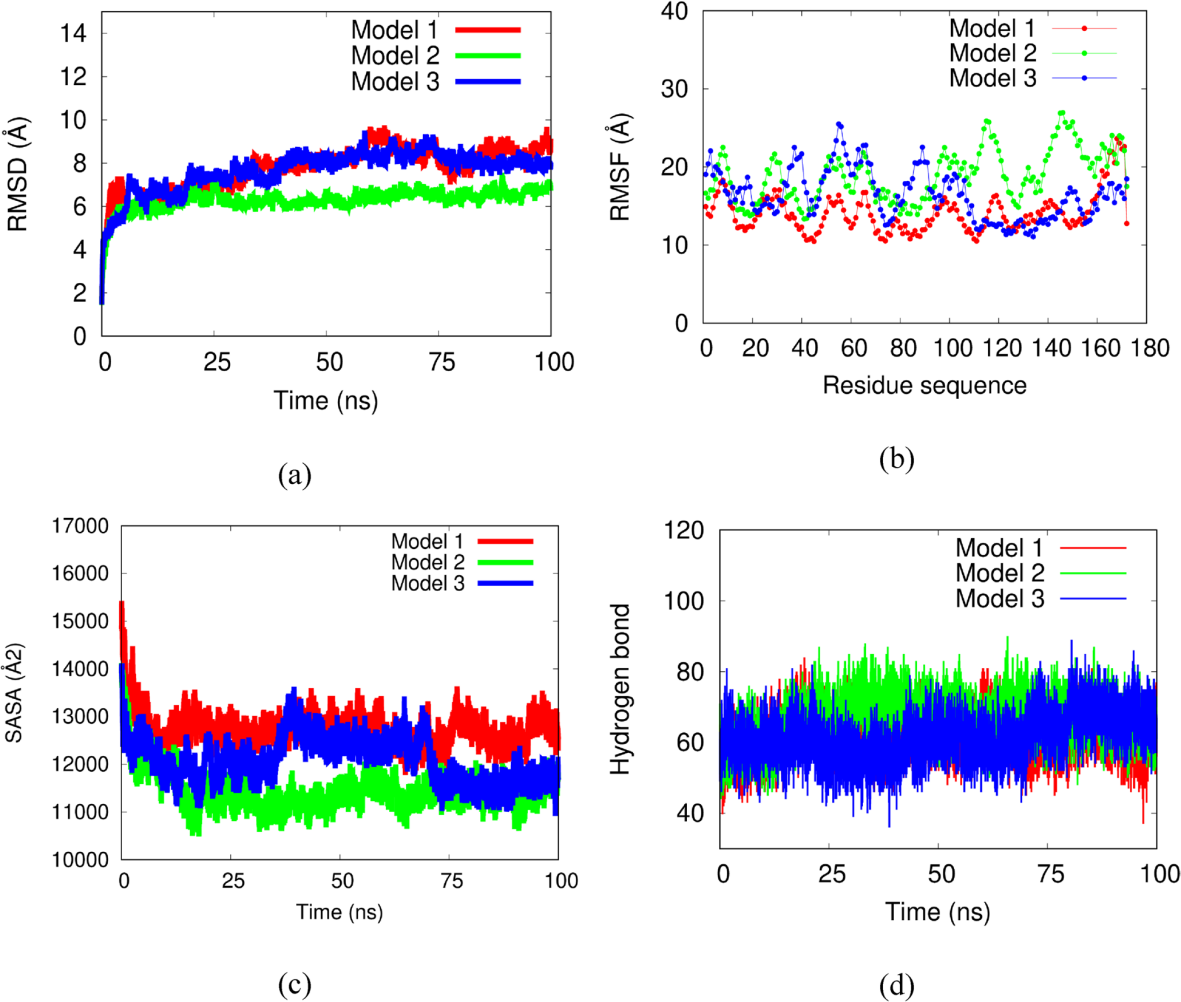
The MD validation metrics consisted of **a** RMSD value of the complexes, **b** RMSF descriptor, **c** protein surface area measured from SASA, and **d** hydrogen bonds

The protein and ligand obtained from docking using the HDOCK web server suggest that the ligand can bind to the receptor site when the complex exhibits negative binding energy. The binding energies obtained from the simulations of the three complexes are listed in Table 3. All ligands are capable of binding to the receptor since all binding energy scores are negative. These results indicate that all ligands can form complexes with the receptor Fig. 4.

**Table 3:** Docking outcomes between the protein fragment Factor C and lipid A utilizing the HDOCK website

**Complex**	**Docking score**	**Confidence score**
**Complex 1 (fragment FC-Core lipid A)**	−467.93	0.9983
**Complex 2 (fragment FC-diphosphorylLipid A)**	−302.86	0.9551
**Complex 3 (fragment FC-Kdo2 lipid A)**	−322.98	0.9695

The docking scores for each complex have negative values with varying binding energy values. In complex 1, the docking score is the highest with a binding energy of −467.93 and a high confidence score of 0.9983. Complex 2 has a docking score of −302.86 and a confidence score of 0.9551, while complex 3 has a docking score of −322.98 and a confidence score of 0.9695. The more negative the binding energy value, the more stable of the ligand-receptor complex formation. This finding suggested that the most stable complex could be found in Complex 1 then followed by Complex 3 and Complex 2, respectively.

### Molecular dynamics simulations of Factor C protein fragment

The parameters of each MD (RMSD, RMSF, SASA, and hydrogen bond) were analyzed to determine the mean value as shown in Table 4.

**Table 4:** Mean values of the parameters of each MD analysis

Complex	RMSD	RMSF	SASA	Hydrogen bond
Complex 1	7.7	14.1	12734.5	61.7
Complex 2	6.3	19	11453	66.4
Complex 3	7.7	16.4	12083.2	61.5

### Construction and cloning of the Factor C gene fragment

**Fig. 5:**
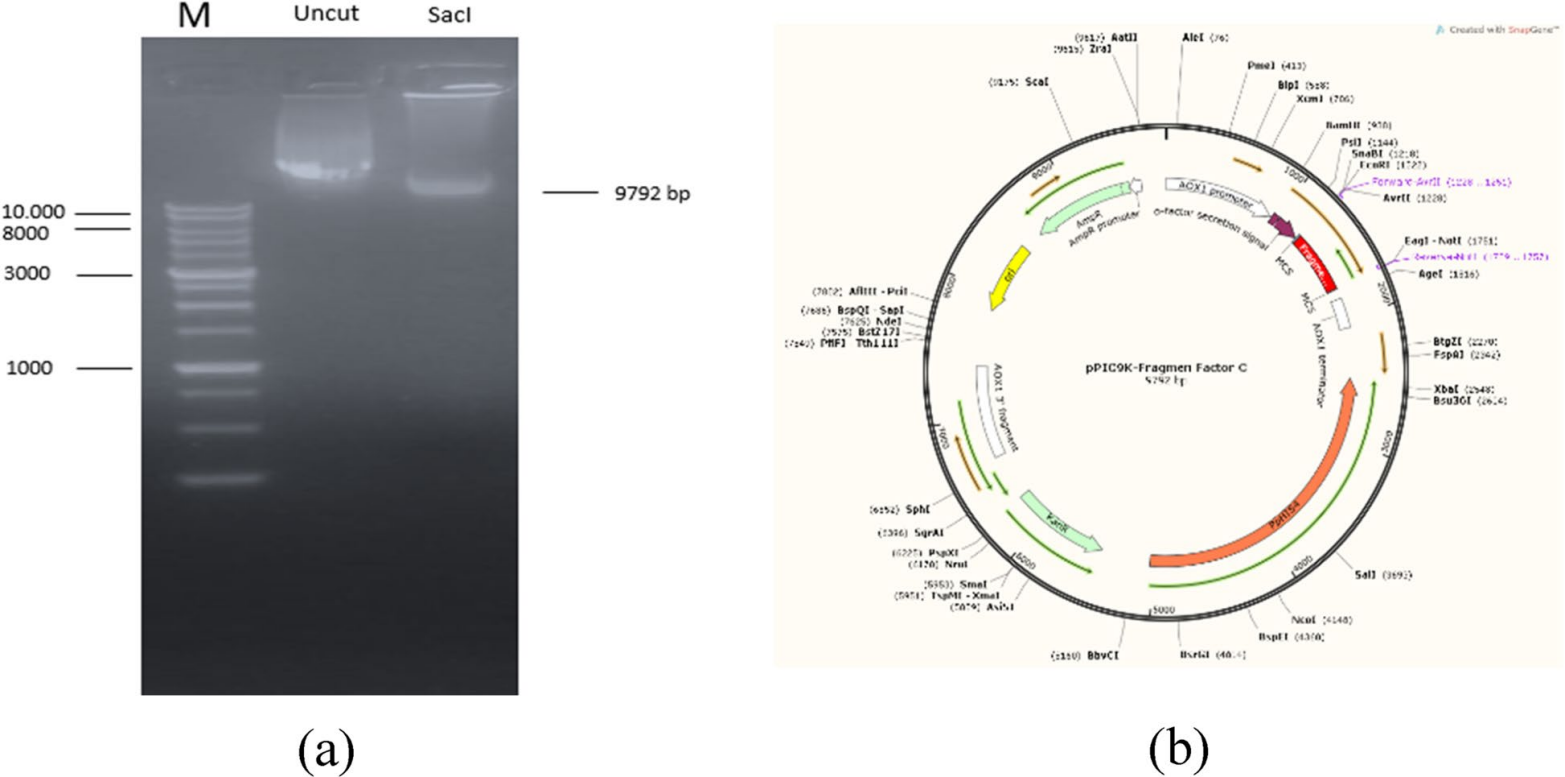
**a** Digestion results of the recombinant plasmid for fragment factor C; lane M = marker, uncut = pPIC9K plasmid - fragment FC, SacI = digestion of the plasmid by the restriction enzyme. **b** Construction of FC gene fragment in pPIC9K

The construction of the Factor C fragment gene and the pPIC9K vector was designed using the SnapGene application, involving the integration of the gene into the pPIC9K vector at the multiple cloning site (MCS). The MCS site was cleaved using AvrII and NotI restriction enzymes, followed by ligating the gene into the vector. The Factor C fragment gene carried by the pPIC9K vector was then sequence-aligned for synthesis. The synthetic gene was amplified through transformation into E. coli DH5α. Successfully cloned synthetic genes were confirmed through plasmid isolation. To verify the presence of the inserted gene in the recombinant plasmid, the isolated plasmid underwent single digestion with restriction enzymes [43]. The pPIC9K vector has a sequence length of 9276 bp, while the Factor C fragment gene comprises a sequence of 516 bp. Gel electrophoresis results showed a thick band with a size of 9792 bp. (Fig. 5B).

**Fig. 6:**
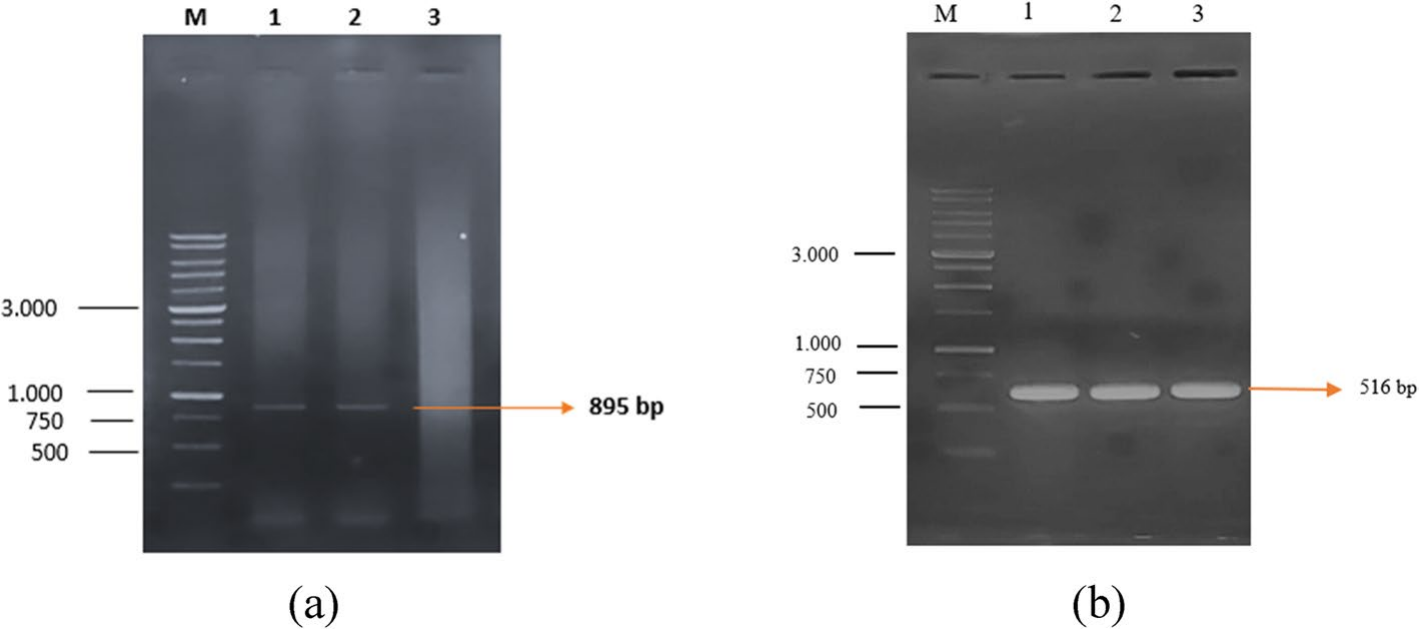
Transformed colony PCR. **a** Primer AOX-forward (pPIC9K) and FC-reverse from Factor C gene fragment. **b** Transformed colony PCR using a pair of FC-forward and FC-reverse from Factor C gene fragment

The results of the single digest of the recombinant pPIC9K vector were transformed on the *P. P. pastoris* (GS115) expression host cells. Furthermore, the success of the transformants was confirmed by employing colony PCR and conducting sequencing analysis with designated primers (Table 5) Fig. 6.

**Table 5:** Information on primer employed

**Primer**	**Utility**	**DNA sequence**	**Annealing (Tm)**	**Amplicon size (bp)**
**AOX-Forward**	Gene amplification	GACTGGTTCCAATTGACAAGC	60	895
**FC-Reverse**		TCTGTCTGCCACTTTAACAC		
**FC-Forward**	Gene amplification	ATGGGCAAGCTTCCAAACTCT	63	516
**FC-Reverse**		TCTGTCTGCCACTTTAACAC

The pPIC9K vector is commonly used as a general expression vector for expression in *P. pastoris*. This expression vector contains the AOX1 nucleotide sequence at the 3' end, the AOX1 promoter sequence at the 5' end, and the HIS4 marker gene [44]. The insert DNA is typically integrated into the *P. pastoris* chromosome to obtain a stable expression system. Integration of the insert DNA into the *P. pastoris* chromosome can be achieved through two strategies: single crossover homologous recombination and double crossover homologous recombination, also known as gene transplacement [45].

### Optimization of Factor C protein fragment expression

**Fig. 7:**
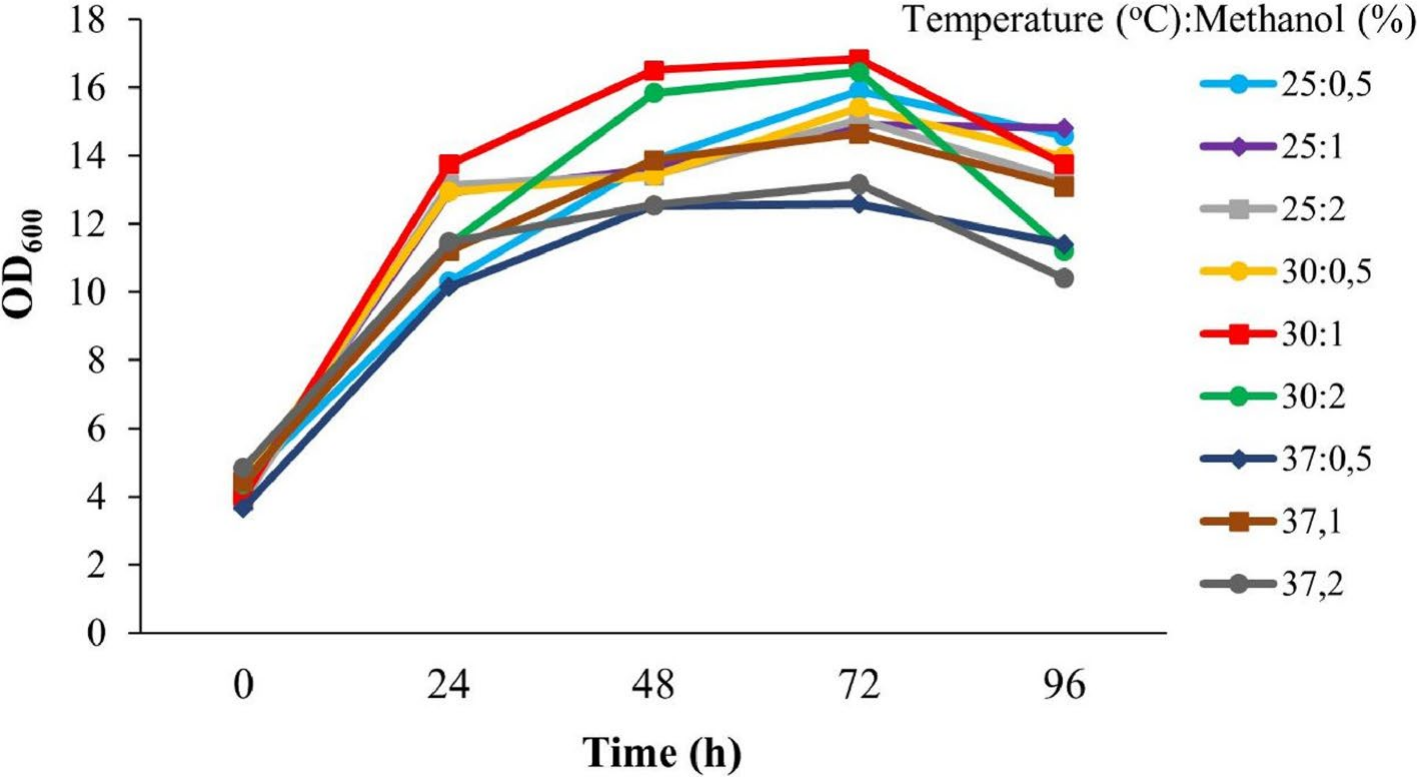
Growth curve of recombinant *P. pastoris* GS115 under optimized MeOH induction and temperature conditions

For the optimization of protein expression of the Factor C fragment, the clone was induced with methanol at concentrations of 0.5%, 1%, and 2%. The induction was performed in a medium with a pH of 6 at various temperatures (25°C, 30°C, and 37ºC). The optimization results showed that the highest OD value of 16.83 was obtained when the induction was carried out with 1% methanol at a temperature of 30°C for 72 h of incubation. This OD value was higher compared to the other treatment conditions (Fig. 7).

As illustrated in Fig. 7, the growth pattern of *P. pastoris* cultured in BMMY media was compared at three different temperature levels. A lower cell density was observed at the end of fermentation when grown at 37°C compared to 25°C and 30°C. This observation supports the findings reported by Li et al. [45], who also observed a decrease in viable cell densities after three days of fermentation at 30°C, compared to 23°C.

**Fig. 8:**
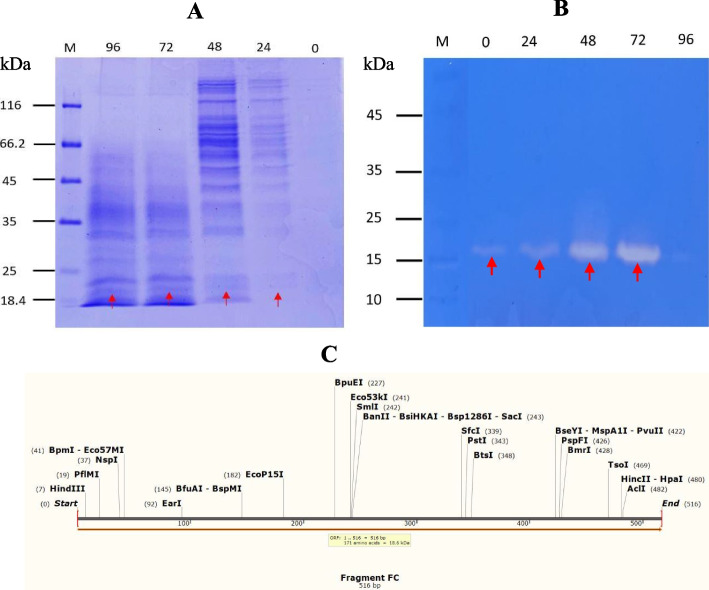
The profile of the Factor C protein fragment from *P. pastoris* GS115. **A** SDS-PAGE analysis of serine protease induction with 1% MeOH at 30°C. M, marker; Lane 0, 0 h (no induction); lane 24, 24 h induction with 1% MeOH; lane 48, 48 h induction with 1% MeOH; lane 72, 72 h induction with 1% MeOH; lane 96, 96 h induction with 1% MeOH. **B** Zymogram analysis of serine protease induction with 1% MeOH at 30°C. M, marker; lane 0, 0 h (no induction); lane 24, 24 h induction with 1% MeOH; lane 48, 48 h induction with 1% MeOH, lane 72, 72 h induction with 1% MeOH, lane 96, 96 h induction with 1% MeOH. **C** Mapping of gene sequence and molecular weight of the Factor C protein (18.6 kDa)

SDS-PAGE and zymogram analyses was employed to measure the weight of the molecular (MW) of the recombinant Factor C protein fragment expressed in *P. pastoris* GS115. The results showed a distinct protein band indicating the MW of the Factor C protein fragment (Fig. 8). Visualization using SDS-PAGE revealed the MW of the Factor C protein fragment to be approximately 18.6 kDa (Fig. 8A). This was further confirmed by zymogram analysis using a casein substrate, which showed that the Factor C protein fragment, belonging to the serine protease group, successfully hydrolyzed the casein substrate when induced with 1% methanol at 30°C for 72 h of fermentation (Fig. 8B).

The *P. pastoris* GS115 expression system, which offers several benefits, was used to express the recombinant protein. This system allows for easy manipulation at the molecular level, post-translational modifications, protein folding, and extracellular protein secretion [46]. The ease of production, relatively inexpensive production equipment, and scalability make the *P. pastoris* expression system highly desirable for producing recombinant proteins [47]. The scalability of the *P. pastoris* expression system allows for larger production compared to the *E. coli* expression system [48]. In some cases, the expression system can achieve higher levels of recombinant protein expression compared to conventional yeast expression systems using *S. cerevisiae* [49].

Methanol was added to the expression medium at final concentrations of 0.5%, 1%, and 2% for the best methanol concentration. The results demonstrated that 1% methanol concentration had the maximum protease activity. At greater methanol concentrations, the protease activity, however, sharply decreased [Fig. 8]. These results are consistent with the cell density measurements taken by monitoring OD_600_ nm every 24 h, which indicated that cell growth reached the logarithmic phase after 72 h of fermentation [Fig. 7]. Subsequently, the expression of the Factor C fragment was tested at 25°C, 30°C, and 37°C to determine the optimal temperature for enzyme production. In our study, we successfully achieved high levels of Factor C fragment protein expression in the methylotrophic yeast *P. pastoris* after 72 h of induction under optimal conditions (30°C and 1% methanol concentration). The Factor C was secreted into the medium at a high level and protease activity was 3254 U/ml [Fig. 8].

## Discussion

Due to its obvious benefits, including as inducible expression utilizing adjustable promoters and the ease of medium conditions, * P. pastoris* has become more popular as a host for heterologous protein production [50]. By employing codon optimization techniques, we achieved successful expression of the Factor C protein fragment at higher levels in this unicellular eukaryotic system. Subsequently, we conducted observations on the solubility of the Factor C protein fragment. Protein solubility is a crucial property in recombinant protein production and biotherapeutics development. Solubility prediction based on protein sequence provides a straightforward approach. The results, presented in Table 3, reveal that the solubility of the Factor C fragment protein is influenced by negatively charged amino acids Asp and Glu, with 6.4% and 4.7% composition, respectively. On the other hand, positively charged amino acids Arg and Lys contribute to the solubility with 5.8% and 5.3% composition, respectively. Additionally, hydrophobic amino acids Val, Leu, and Ile account for 8.2%, 7.6%, and 3.5%, respectively, while aromatic amino acids Phe, Tyr, and Trp contribute 4.7%, 2.3%, and 2.9%, respectively. Aromatic residues and hydrophobic interactions have a minor impact on protein aggregation formation. Further confirmation through the Protein-Sol webserver yielded a solubility value of 0.454. The Protein-Sol analysis relies on the Population average Solubility (PopAvrSol) value, where a value greater than 0.45 indicates higher solubility, while values below 0.45 suggest lower solubility [51].

Molecular docking is a method used to examine how tiny molecules behave inside a protein’s binding pocket and to forecast the potential affinities during binding [52]. Lipopolysaccharide (LPS) consists of three main components: O antigen, lipid A, and core oligosaccharide. Factor C recognizes LPS based on endotoxin activity. Among the three components of LPS, lipid A is considered the main constituent and plays a crucial role. Therefore, docking studies between Factor C and lipid A were performed to explore the binding site activity. Lipid A is of particular importance as it exhibits strong endotoxin detection capabilities. By binding to the receptor’s area to create a ligand-receptor complex, three ligands diphosphoryl lipid A, core lipid A, and Kdo2 lipid A could behave as activators of the Factor C protein, according to our docking simulation. As a result, three structures (Complexes 2, 3, and 4) were chosen for additional examination [25].

In the previous results, 3 models (core lipid A, diphosphoryl lipid A, and Kdo2 lipid A) were identified by using molecular docking. We then performed MD simulation on these three complexes to assess their stability in water solvent. Four validation metrics including root mean square deviation (RMSD), root mean square deviation (RMSF), solvent accessible surface area (SASA), and hydrogen bonds were calculated for all models. Figure 4a showed the RMSD as a function of time for all models. In this figure, all models were involved large fluctuation at 0−5 ns, but after this time, the RMSD value reached the stable state along the simulation. The small fluctuation during simulation is usually found at the protein-ligand complex due to some interactions in the system such as hydrogen bonds, hydrophobic interaction, electrostatic interaction, and water molecules. These interactions contribute to the structural rearrangement protein/ligand complex to achieve the equilibrium state. From the RMSD graph trends, all complexes were stable during the simulation. To know the flexibility of protein-ligand complex, RMSF profile was estimated. Models 2 and 3 seem to be more flexible as provided in Fig. 4b, suggesting the ligands could be easily bound to the site of proteins. In Fig. 4c, SASA value was calculated for all models. SASA profile is used to know the volume of the protein-ligand complex along the simulation. All complexes are not significant difference of SASA, indicating all protein-ligand complex formation might be stable during the simulations. Furthermore, hydrogen bonds were analyzed for all models. This interaction was also involved to maintain the structural interface of the protein-ligand. Figure 4d provided the hydrogen bond ranging from 40–80 for all complexes and the graphic trends tends to similar form. This finding suggested that hydrogen bond contributed in holding a stable structure of protein-ligand complex during the MD simulation.

The expression of Factor C from * T. gigas* using *P*. *pastoris* (GS115) as the host cell is reported here for the first time. In the case of producing different recombinant proteins, the Pichia expression system has several advantages [53]. Since *P. pastoris* produces less endogenous secretory proteins in this expression system, it is simpler to purify recombinant proteins [54]. To maximize the expression of the Factor C fragment, the time course of methanol-induced peptide synthesis was adjusted in this investigation. We employed AOX1, a potent methanol-induced promoter, and examined the effects of various methanol concentrations on the expression of heterologous proteins. The primary carbon source and gene expression inducer in the majority of *P. pastoris* fermentation techniques is methanol. Our findings demonstrated that increased cell growth of *P. pastoris* was seen when treated with 1% methanol in BMMY medium and incubated at 30°C for 72 h; higher cell growth of *P*. *pastoris* was observed (OD_600_=16.83). The production of recombinant proteins in *P. pastoris* is known to be influenced by culture and induction conditions [55–57]. The concentration of methanol, as an inducer, directly affects protein production due to the use of an inducible promoter [58]. While low levels of methanol may not effectively induce the AOX1 promoter, excessive amounts of methanol can be toxic to the cells. Protease activity was found to significantly decrease at higher methanol concentrations as the inducer.

**Fig. 9:**
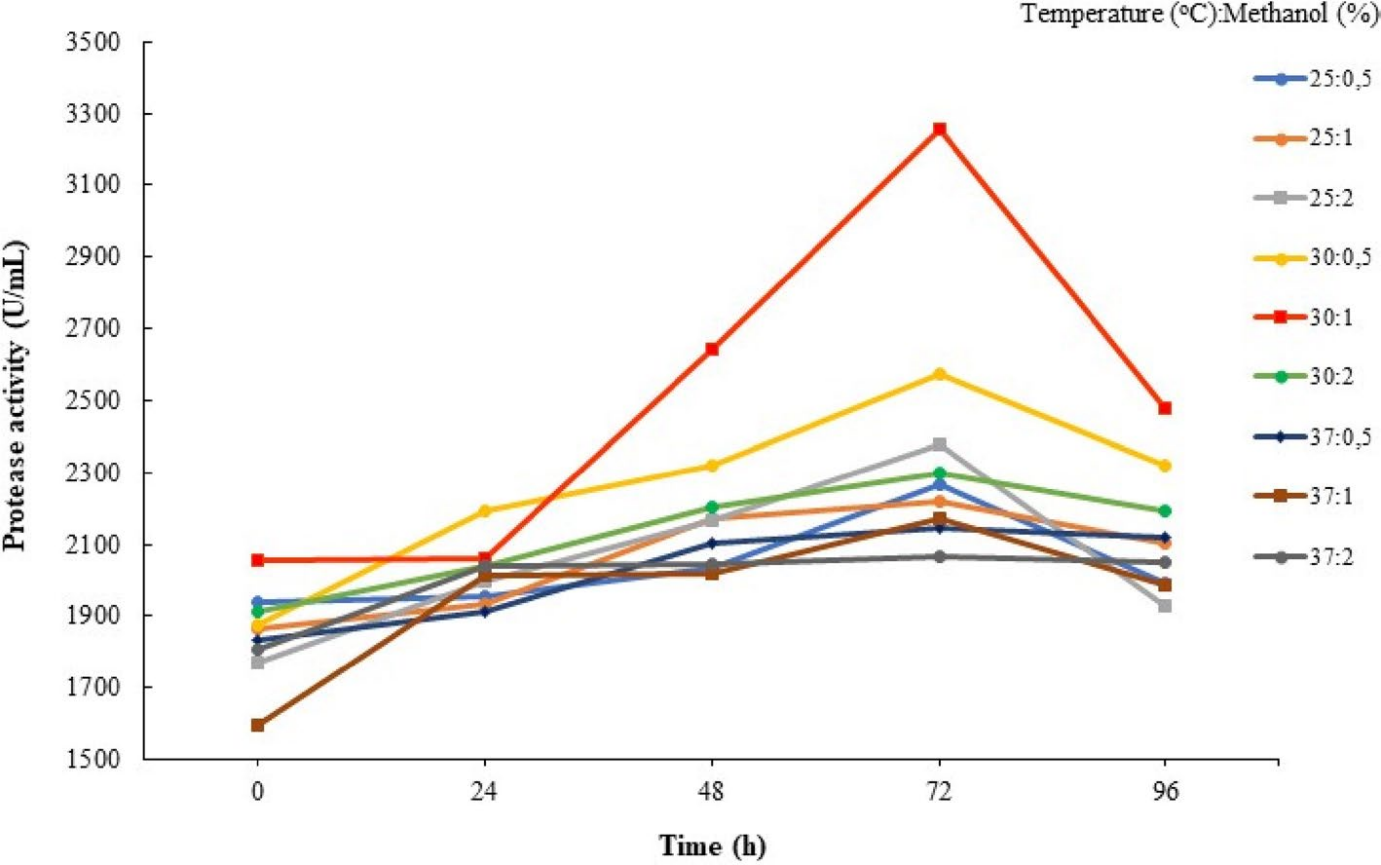
The Profile of protease activity of recombinant *P. pastoris* GS115 expressing the Factor C protein after induction with varying concentrations of MeOH at different temperatures

Following the optimization of the Factor C fragment gene and cultivation of recombinant *P. pastoris* GS115, we successfully detected the 18.6 kDa Factor C fragment protein using SDS-PAGE and zymogram techniques. We report that the Factor C fragment consists of 516 bp, which translates to 171 amino acids. Based on the translation, the calculated molecular weight of the Factor C protein is 18.6 kDa [Fig. 9C]. The appropriate methanol concentration varied between 0.1 and 3% (v/v) [59] based on different foreign proteins. The alcohol oxidase (AOX) enzyme is encoded by two genes in the *P. pastoris* GS115 strain (AOX1 and AOX2). Methanol induces the transcription of these genes, which leads to the production of a significant quantity of AOX enzyme [60]. Methanol cannot efficiently stimulate the AOX1 promoter at low concentrations, but excessive levels of methanol can be harmful to cells [61]. The growth of *P. pastoris* GS115 cells exhibited a significant decline at higher concentrations of methanol used as an inducer (Fig. 7). Higher methanol concentrations appear to promote misfolded proteins while also accelerating cell growth and protein expression [62]. Protein concentration and activity rose at the highest concentration of methanol (2%), probably as a result of cell death and the subsequent release of intracellular proteins in medium culture.

According to various protocols, the optimal temperature for *P. pastoris* cell growth is 30°C. In this study, maximum cell growth of *P. pastoris* GS115 was also observed at 30°C (Fig. 7). An increase in cell numbers was also observed at 25°C after 72 h of induction with 0.5% methanol. Similarly, Naseem et al. [63] reported that maximum expression of rMgTx was achieved when biomass was induced with 0.5% methanol. Due to the sensitivity of *P. pastoris* cells to temperature, high temperatures can negatively impact cell growth and protein expression. Since the recombinant Factor C fragment from *P. pastoris* is intended for industrial-scale applications, a relatively fast protein expression time is desirable. Therefore, the recommended expression time is 72 h of induction with 1% methanol at 30°C.

## Conclusions

Protein binding in several complexes there are amino acid residues that bind in the same region to the amino acid Tyr46 so that it can be estimated that recombinant protein fragment Factor C has activity. Molecular dynamics (MD) simulations were conducted on all three complexes to evaluate their stability in water solvent. The results showed that all complexes are stable along the simulation. We have also successfully demonstrated the expression of the Factor C gene fragment of * T. gigas* in the eukaryotic organism P. pastoris, as a vector system. Based on our findings, we recommend optimizing the expression of Factor C gene fragments by inducing them with 1% methanol at a temperature of 30°C and incubating them for 72 h. This optimized condition can be used for the production of recombinant Factor C gene fragments on a larger scale in a biofermentor.

## Data Availability

Not applicable
